# Emilin2 marks the target region for mesenchymal cell accumulation in bone regeneration

**DOI:** 10.1186/s41232-024-00341-6

**Published:** 2024-06-03

**Authors:** Yifan Qing, Takehito Ono, Yukihiro Kohara, Atsushi Watanabe, Noboru Ogiso, Masako Ito, Tomoki Nakashima, Sunao Takeshita

**Affiliations:** 1 Department of Cell Signaling, Graduate School of Medical and Dental Sciences, Tokyo Medical and Dental University, 1-5-45, Yushima, Bunkyo-Ku, Tokyo 113-8549 Japan; 2 Laboratory of Drug Discovery and Pharmacology, Faculty of Veterinary Medicine, Okayama University of Science, 1-3 Ikoino-Oka, Imabari-Shi, Ehime, 794-8555 Japan; 3 Department of Bone and Joint Disease, National Center for Geriatrics and Gerontology, 7-430, Morioka-Cho, Obu, Aichi Prefecture 474-8511 Japan; 4 Department of Molecular Pharmacology, Graduate School of Pharmaceutical Sciences and Faculty of Pharmaceutical Science, Tokyo University of Science, 2641 Yamazaki, Noda, Chiba 287-8510 Japan; 5 Equipment Management Division, Center for Core Facility Administration, National Center for Geriatrics and Gerontology, 7-430, Morioka-Cho, Obu, Aichi Prefecture 474-8511 Japan; 6 Laboratory of Experimental Animal, Center for Core Facility Administration, National Center for Geriatrics and Gerontology, 7-430, Morioka-Cho, Obu, Aichi Prefecture 474-8511 Japan; 7 Nagasaki University, 1-14, Bunkyomachi, Nagasaki 852-8521 Japan; 8 Faculty of Dentistry, Tokyo Medical and Dental University, 1-5-45, Yushima, Bunkyo-Ku, Tokyo 113-8549 Japan; 9 Aging Stress Response Research Project Team, National Center for Geriatrics and Gerontology, 7-430, Morioka-Cho, Obu, Aichi Prefecture 474-8511 Japan; 10 Angitia Biopharmaceuticals, 2F, Unit 2, Building4, 188 Kaiyuan Avenue, Huangpu District, Guangzhou, 510530 China

**Keywords:** Bone, Regeneration, Mesenchymal cells, Macrophages

## Abstract

**Background:**

Regeneration of injured tissue is dependent on stem/progenitor cells, which can undergo proliferation and maturation processes to replace the lost cells and extracellular matrix (ECM). Bone has a higher regenerative capacity than other tissues, with abundant mesenchymal progenitor cells in the bone marrow, periosteum, and surrounding muscle. However, the treatment of bone fractures is not always successful; a marked number of clinical case reports have described nonunion or delayed healing for various reasons. Supplementation of exogenous stem cells by stem cell therapy is anticipated to improve treatment outcomes; however, there are several drawbacks including the need for special devices for the expansion of stem cells outside the body, low rate of cell viability in the body after transplantation, and oncological complications. The use of endogenous stem/progenitor cells, instead of exogenous cells, would be a possible solution, but it is unclear how these cells migrate towards the injury site.

**Methods:**

The chemoattractant capacity of the elastin microfibril interface located protein 2 (Emilin2), generated by macrophages, was identified by the migration assay and LC–MS/MS. The functions of Emilin2 in bone regeneration were further studied using *Emilin2*^–/–^ mice.

**Results:**

The results show that in response to bone injury, there was an increase in Emilin2, an ECM protein. Produced by macrophages, Emilin2 exhibited chemoattractant properties towards mesenchymal cells. *Emilin2*^–/–^ mice underwent delayed bone regeneration, with a decrease in mesenchymal cells after injury. Local administration of recombinant Emilin2 protein enhanced bone regeneration.

**Conclusion:**

Emilin2 plays a crucial role in bone regeneration by increasing mesenchymal cells. Therefore, Emilin2 can be used for the treatment of bone fracture by recruiting endogenous progenitor cells.

**Supplementary Information:**

The online version contains supplementary material available at 10.1186/s41232-024-00341-6.

## Introduction

Biological tissues are subjected to various environmental stimuli. Intense stimuli can cause tissue damage. Damaged tissues undergo regeneration, which is dependent on stem/progenitor cells. The proper functions of these cells are crucial for recovering the structure and function of the damaged tissues [1–3]. The bone is a highly regenerative tissue that recovers its structure and functions without scar formation after fracture. Fractured bone regenerates through a sequence of processes that consists of hematoma formation, inflammation, callus formation, and remodeling [4, 5]. Mesenchymal progenitor cells, which are found in the bone marrow, periosteum, and muscles, play a central role in callus formation to reestablish the bony continuity by differentiating into fibroblasts, chondrocytes, and osteoblasts [6–9]. A decrease in these progenitor cells under certain conditions (e.g., aging and radiation) leads to delayed healing or failure in bone regeneration (nonunion), while stem cell transplantation has been shown to promote regeneration in animal studies [10, 11].

Currently, stem cell therapy has garnered the attention of clinicians, and many clinical studies are underway. Such therapies are expected to improve the treatment outcomes but there are also drawbacks such as the accumulation of stem cells in the lung after intravenous injection, immune responses against allogenic stem cells, low rate of cell viability, oncological complications, and a need for special devices for cell expansion [5, 12, 13]. The employment of endogenous stem cells would be another option for treating bone fractures. The migration of mesenchymal stem/progenitor cells to the injury site can enhance bone regeneration. It has been reported that C–X–C motif chemokine 12 (CXCL12) and its receptor C–X–C chemokine receptor type 4 (CXCR4) are involved in bone regeneration by facilitating chemoattraction [14–16]. However, the mechanism by which mesenchymal cells are recruited to the injury site after bone fracture remains unclear.

Macrophages, which are myeloid immune cells, were first discovered due to their fundamental role in phagocytosis and clearance of microorganisms. They were subsequently found to have a variety of functions including tissue maintenance/destruction, metabolic homeostasis/disorders, and cancer suppression/progression. The complex nature of these cells is attributed to the complex process of their development and plasticity [17]. Given rise to in the Yolk sac or the bone marrow, and polarized to gain tissue-specific or disease-specific phenotypes, macrophages produce a wide range of cytokines and chemokines. During the process of bone regeneration, they produce cytokines, promote angiogenesis, and differentiate into osteoclasts [18–21], coupling bone injury and regeneration processes. Despite their property in producing various chemoattractants, it remains unclear if macrophages contribute to mesenchymal cell recruitment in bone regeneration.

Here, we found that Emilin2, a component of extracellular matrix (ECM) protein, is a chemoattractant produced by macrophages. Emilin2 is produced and deposited at the bone injury site. *Emilin2*^–/–^ mice have impaired bone regeneration, with decreased mesenchymal cells. Local administration of recombinant Emilin2 protein enhances bone regeneration. Together, these data indicate that Emilin2 is produced by macrophages and deposited at the site of injury to mark the target region for mesenchymal cell accumulation. Thus, mesenchymal cells migrate and differentiate into osteoblasts to form the callus, facilitating bone regeneration. To treat bone fracture, Emilin2 can be implanted at the injury site to recruit endogenous progenitor cells.

## Materials and methods

### Experimental animals

Seven-week-old C57BL/6N male mice were purchased from Clea Japan and underwent acclimatization for a week before experiments. *Emilin2*^–/–^ mice were generated as described in Additional file 3: Fig. S3a–d. In detail, all-in-one ready-to-use Cas9 and gRNA expression plasmid (Sigma-Aldrich, Target ID; MM0000512698 for mouse *Emilin2*, USA) was microinjected into pronuclei of C57BL/6N zygotes. To minimize the risk of off-target effects, the founder mouse was backcrossed with C57BL/6N mice. The second filial generation (F2) and later generation of mice were used for analyses. For sequencing of *Emilin2* mutation, the primer with the sequence of 5′-AAGTGAATTAAAGAGGGCGG-3′ was used. For genotyping PCR, the following sequences of primers were used: amplification, 5′-AATAACTGATGGGTGGTCC-3′ and 5′-GAGCATAAGGGACACACTCT-3′; wild-type allele detection, 5′-GGCGATGGGCCGAGAGATG-3′ and 5′-CGACTGGGTGCTCGGGGT-3′; and knockout allele detection, 5′-GGCGATGGGCCGAGAGATG-3′ and 5′-CGACTGGGTGCTCGGGGC-3′.

*Emilin2*^–/–^ mice were generated and all experiments were performed in accordance with the National Center for Geriatrics and Gerontology (NCGG) ethical guidelines for animal care, and the study protocol was approved by the Animal Care Committee. These mice were also maintained at Tokyo Medical and Dental University under specific pathogen-free conditions. All the animal experiments were approved by the Institutional Animal Care and Use Committee and Genetically Modified Organisms Safety Committee of Tokyo Medical and Dental University (approval No. A2023-026C and G2023-011C, respectively) and conducted in accordance with the guidelines concerning the management and handling of experimental animals.

### Mouse models

Drill-hole injury was performed as previously described [6, 22]. In brief, mice were anesthetized with an intraperitoneal injection of a mixture of the following solution: midazolam (1.6 mg·mL^–1^), medetomidine hydrochloride (0.06 mg·mL^–1^), and butorphanol tartrate (1 mg·mL^–1^) in normal saline. Five microliters of·gBW^–1^ was intraperitoneally injected. The skin of the right thigh was exposed by hair removal, after which disinfection using ethanol was conducted. The surface of the femur was exposed by incision of the skin and splitting the muscle immediately above the femur. After the removal of the periosteum, a drill hole with a diameter of about 0.8–1.2 mm was made on the anterior portion of the diaphysis of the femur. The incised muscles and skin were closed with nylon sutures.

Transplantation of drilled diaphyses of *Emilin2*^+/+^ and *Emilin2*^–/–^ mice was performed as previously described [14]. Briefly, after the right femoral drill hole was created in a donor mouse, a 25G needle was punctured from the distal to the proximal end of the femur for stabilization. A dental finishing disc was applied to partly transect the femur approximately 2 mm proximal and distal to the drill hole, and then after removing the 25G needle for stabilization, the scissors were utilized to resect the diaphysis part with a drill hole. The resected femoral diaphysis was transplanted into the femoral diaphysis site of the recipient mouse, which had received the same procedure. Another 25G needle was inserted through the distal to proximal ends for fixation. The incised muscles and skin were closed with nylon sutures.

Macrophage depletion was conducted using clodronate-liposomes. After creating drill holes, 25 µL clodronate-liposomes (Hygiea Biosciences, India) or empty-liposomes (Hygiea Biosciences) were applied around the defect.

To obtain recombinant mouse Emilin2-His (rEmilin2-His) protein, mouse *Emilin2* cDNA containing a His-tag in the pCXN2 vector [23] was transfected into mouse fibroblastic Ltk^−^ cells using X-tremeGENE 9 DNA Transfection Reagent (Roche Diagnostics, Switzerland). After selection with G418 (400 mg·mL^–1^) (Gibco Life Technologies Corp., USA), clones producing high Emilin2 levels were selected by limiting dilution followed by reverse transcriptase-polymerase chain reaction (RT-PCR) for *Emilin2* mRNA expression. Cell culture supernatant was harvested, and rEmilin2-His protein was purified using HisTrap HP (GE Healthcare, USA) and Amicon Ultra-15 (Millipore Corporation, USA). All other reagents were from Sigma Chemical Co. Recombinant Emilin2 was administrated using a carrier. Ten microliters of recombinant Emilin2 (1.55 µg·µL^–1^) or phosphate-buffered saline (PBS) was immersed into a controlled-release Medgel® Sheet II (PI5) (Nitta Gelatin Inc., Japan) block overnight at 4°C. The block was placed on the drill hole and was covered by sutured soft tissues. The loading concentration was determined higher than that used in the in vitro experiments taking into account the controlled release property of the scaffold and the processes of drug metabolism and excretion in vivo.

### Tissue harvest and preparation

For bone histomorphometric analyses, mice underwent double labeling with tetracycline and calcein was performed by consecutive administration of the labels subcutaneously with a 2-day interval [24]. Bone samples for micro-computed tomography (CT) analyses and histomorphometric analyses were fixed with 70% ethanol at 4 °C. Tissue samples for histological and immunohistological analyses were fixed using 4% paraformaldehyde (PFA) at 4 °C overnight.

Bone marrow cells were extracted from the femur and tibia by introducing PBS into the bone marrow cavity. These cells were used for chemotaxis assay and osteoclast differentiation experiment, which are described below.

For flow cytometric analysis and in vitro osteoblast differentiation, tissues in the drill hole, periosteum, and skeletal muscle were digested in a collagenase (WAKO, Japan) solution (1 mg·mL^–1^). After debris removal by filtration using 70 or 40 μm mesh (Corning, USA), the collected cells underwent hemolysis [6].

For RNA extraction, tissues were minced with scissors, lysed in Sepasol®-RNA I Super G (Nacalai Tesque, Japan), and stored at –80 °C before synthesizing complementary DNA (cDNA).

### Micro-CT analysis

For the analysis of cancellous bone of *Emilin2*^+/+^ and *Emilin2*^–/–^ mice, micro-CT scanning was performed using a μCT-40 device (SCANCO Medical, Switzerland) at an isotropic voxel size of 12 μm, and 3D microstructural parameters were calculated as described previously [24]. The nomenclature for micro-CT followed the recommendations of the published guideline [25].

For the evaluation of bone regeneration in the drill hole, micro-CT scanning was conducted using a ScanXmate-A100S Scanner (Comscantechno, Japan) with the following parameters: voltage, 75 kV; current, 140 μA; and isotropic voxel size, 7 μm. A three-dimensional microstructural data was obtained using TRI/3D-BON software (Ratoc System Engineering, Japan).

### Histomorphometric analysis of the bone

Bone samples fixed in 70% ethanol were embedded in glycol methacrylate (GMA), and non-decalcified sections were made. Histomorphometric parameters were analyzed at the Ito Bone Science Institute (Niigata, Japan). The nomenclature for histomorphometry followed the recommendations of the published guideline [26].

### Histological analysis and immunohistological analyses (paraffin section)

The fixed samples underwent decalcification using an ethylenediaminetetraacetic acid (EDTA) solution, EDT-X (FALMA, Japan) for 21 days. Samples were further dehydrated and embedded in paraffin. Six-micrometer-thick sections were cut. After deparaffinization and hydration, the sections underwent staining.

Tartrate-resistant acid phosphatase (TRAP) staining was performed using a staining solution of the following constitution: 0.5 mg·mL^–1^ Naphthol AS-BI phosphate sodium salt, 1.2 mg·mL^–1^ sodium nitrite, 1.2 mg·mL^–1^ pararosaniline hydrochloride, and 8.3 mg·mL^–1^ disodium tartrate dihydrate in 0.1 M acetic acid-sodium acetate buffer [pH 5.1]. Tissues were stained for 12 min at room temperature. Nucleus counterstaining was conducted by hematoxylin (Muto Pure Chemicals, Japan) [6].

For immunohistological analyses, the sections underwent an antigen retrieval in a Tris–EDTA buffer [pH 9.0] overnight at 55°C. After the retrieval, the sections were incubated with the following primary antibodies at room temperature (RT): rabbit anti-Emilin2 (1/100, Cloud Clone, USA) for 120 min and rabbit anti-Osterix (1/1000, Abcam, UK) for 60 min. Secondary antibodies for visualization using horseradish peroxidase and 3,3′-diaminobenzidine (DAB) system or fluorescence by donkey anti-rabbit IgG Alexa Fluor 488 (1/500, Invitrogen, USA). Nucleus counterstaining was conducted by hematoxylin (for DAB staining) or Hoechst33342 (for immunofluorescence). The microscopic images were obtained and analyzed using a microscope and measurement software (BZ700 and BZ-X analyzer, Keyence, Japan).

### Flow cytometry

Cells harvested from the mice immediately underwent flow cytometric analysis. Nonspecific binding was blocked with anti-cluster of differentiation 16/32 (CD16/32) (93, BioLegend, USA) at a dilution of 1/100. The antibodies used for flow cytometric analysis were as follows. Anti-mouse CD8α (53–6.7, BioLegend), CD31 (390, BioLegend), TER-119 (TER-119, BioLegend), CD45 (30-F11, BioLegend), Sca-1 (D7, BioLegend), CD140α (APA5, BioLegend), and CD11b (M1/70, BioLegend). These antibodies were used at a dilution of 1/250. Dead cells were stained using 7-amino-actinomycin D (7-AAD) (BioLegend) before analysis at a dilution of 1/41. Data were acquired on a flow cytometer, CytoFLEX S (BECKMAN COULTER, USA), and analyzed using FlowJo software (TREE STAR, USA).

### Protein purification from the supernatant of cultured macrophages

Bone marrow macrophages were cultured in *α*-minimum essential medium (MEM) containing 10% fetal bovine serum (FBS) and penicillin/streptomycin (Gibco, USA) in the presence of macrophage colony-stimulating factor (M-CSF) (10% CMG14-12 culture supernatant) for 3 days as described previously [27]. Cells were washed twice with PBS and were further cultured in *α*-MEM in the presence of M-CSF without FBS for 3 days. Conditioned medium was collected and concentrated with buffer exchanged into a 10-mM HEPES buffer (pH 6.0, buffer 1) using Pericon XL Filter 10 K (Millipore Corporation). Conditioned medium was applied to a HiPrep Q Sepharose FF 16/10 (GE Healthcare) equilibrated with 20-mM Tris–HCl (pH8.0, buffer 1). After washing with a 5-column volume (100 ml) of buffer 1, bound proteins were eluted by a gradient of 0 to 100% 1 M NaCl in buffer 1. The column eluate with highly concentrated chemotactic activity was dissolved in sodium dodecyl sulfate–polyacrylamide gel electrophoresis (SDS-PAGE) sample buffer and was fractioned by SDS-PAGE in 5–20% acrylamide gels. The gels were then stained using Ez Stain Silver (ATTO, Japan), and major protein bands were excised with a scalpel and analyzed by mass spectrometry.

### Cell migration assay

Chemotaxis of mesenchymal cells was assessed using the Cultrex 96 Well Cell Migration Assay Kit (Trevigen Inc., USA) as described previously [24]. In brief, ST2 cells at 80% confluence were serum starved in the 0.5% FBS for 24 h, then harvested and added to the top chamber at the concentration of 5 × 10^4^ cells per well. The conditioned medium of bone marrow cells or the medium including 0 or 10 μg/ml of recombinant Emilin2 was added to the bottom chamber and incubated for 24 h. Migrating cells were stained with calcein and examined with a reader plate at 485 nm excitation and 520 nm emission.

### Mass spectrometry analysis

The fractions possessing mesenchymal cell migrating activity were added in the SDS-PAGE sample buffer and fractionated by SDS-PAGE in 5–20% acrylamide gels. After staining with Coomassie brilliant blue, all protein bands were excised with a scalpel and analyzed by mass spectrometry.

Proteins in the excised gels were reduced with 10-mM dithiothreitol at 65 °C for 1 h and alkylated with 40 mM iodoacetamide in the dark at room temperature for 30 min. Each sample was digested with sequencing-grade modified trypsin (4 μg·mL^–1^) (Trypsin Gold, Promega, USA) in 40 mM NH_4_HCO_3_/10% ACN at 37 °C overnight. The extracted peptides were then separated by nano-flow liquid chromatography (LC) (Paradigm MS4, Michrom BioResources, Inc., USA) using a reverse phase C18 column (Magic C18, 0.2 × 50 mm; Michrom BioResources, Inc.). The LC eluent was coupled to a micro-ionspray source attached to an LCQ Advantage MAX mass spectrometer (Thermo Fisher Scientific, USA). All MS/MS spectra were searched using the TurboSEQUEST algorithm within the BioWorks 3.2 software (Thermo Fisher Scientific), and the data were submitted to the MASCOT program for identification of mesenchymal cell-attracting proteins.

### In vitro osteoblast differentiation

Cells harvested from the mice were immediately underwent magnetic activated cell sorting (MACS) and in vitro culture. Nonspecific binding was blocked with anti-CD16/32 (93, BioLegend) at a dilution of 1/100. The cells were incubated with the following antibodies at a dilution of 1/200: CD31 (390, BioLegend), TER-119 (TER-119, BioLegend), and CD45 (30-F11, BioLegend). For indirect magnetic labeling were anti-phycoerythrin (PE) MicroBeads (Miltenyi Biotec, Germany) at a dilution of 1/5. After flowing through the MS column (Miltenyi Biotec) on the OctoMACS™ Separator (Miltenyi Biotec), the cells negative for CD31, TER-119, and CD45 were collected and seeded on the 48-well plate at the concentration of 1.4 × 10^4^ cells per well with an osteogenic medium containing 10% FBS, 50 μg·mL^–1^ ascorbic acid, 10 nmol·L^–1^ dexamethasone, and 10 mmol·L^–1^ β-glycerophosphate in *α*-MEM. The differentiation medium was changed every third day. Recombinant Emilin2 was added on days of the medium change, at the concentration of 0, 2, or 10 μg·mL^–1^, which was determined based on the result in our migration assay (see “Cell migration assay” section).

Alkaline phosphatase (ALP) staining was performed as follows on day 14. After fixation with 4% PFA for 20 min., these cells were stained with a staining solution of the following components: Napthol AS-MX phosphate, 0.06 mg·mL^–1^; *N*,*N*-dimethylformamide, 1%; and Fast blue BB salt, 1 mg·mL^–1^ in 0.1 mmol·L^–1^ Tris–HCl [pH 8.0]. The stained images were obtained under microscopy (BZ-X analyzer, Keyence). The total RNA was extracted from these cells using Sepasol®-RNA I Super G (Nacalai Tesque) and stored at –80°C before synthesizing cDNA.

### In vitro osteoclast differentiation from mouse bone marrow cells

Bone marrow cells extracted from the femur and tibia were seeded on the 24-well plate at the concentration of 1.0 × 10^5^ cells per well. These cells were expanded in *α*-MEM with 10 ng·mL^–1^ M-CSF (R&D systems, USA) for 2 days, then stimulated with differentiation medium containing 10 ng·mL^–1^ M-CSF and 25 ng·mL^–1^ receptor activator of nuclear factor kappa-Β ligand (RANKL) (PeproTech, USA) in *α*-MEM (day 0). The differentiation medium was changed on day 2. On day 3, osteoclasts were detected by TRAP staining as previously described [28, 29].

### Quantitative RT-PCR (qRT-PCR)

The total RNA extracted from mouse tissues and cultured cells underwent cDNA synthesis using ReverTra Ace® (TOYOBO, Japan). cDNA thus synthesized was subjected to qRT-PCR analysis. PCR reaction was carried out using SYBR Green Real-time PCR Master Mix (TOYOBO) or THUNDERBIRD Next SYBR qPCR Mix (TOYOBO) and a Light Cycler apparatus (Bio-Rad Laboratories, USA). ΔΔCt method was adopted for calculating gene expression and glyceraldehyde-3-phosphate dehydrogenase (*Gapdh*) expression was used for normalization. The primer sequences are listed below: *Gapdh*, 5′-ACCCAGAAGACTGTGGATGG-3′ and 5′-CACATTGGGGGTAGGAACAC-3′; bone γ-carboxyglutamate protein (*Bglap*), 5′-GCGCTCTGTCTCTCTGACCT-3′ and 5′-ACCTTATTGCCCTCCTGCTT-3′; alkaline phosphatase (*Alpl*), 5′-AACCCAGACACAAGCATTCC-3′, and 5′-GCCTTTGAGGTTTTTGGTCA-3′; Sp7 transcription factor (*Sp7*), 5′-ACTGGCTAGGTGGTGGTCAG-3′, and 5′-GGTAGGGAGCTGGGTTAAGG-3′; runt-related transcription factor 2 (*Runx2*), 5′-CCCAGCCACCTTTACCTACA-3′ and 5′-TATGGAGTGCTGCTGGTCTG-3′; *Emilin2*, 5′-CCCTGCTACCAAGGTGGATG-3′ and 5′-TTGACGCTCCCTGTCTTTGC-3′; epidermal growth factor receptor (*Egfr*), 5′-ACACTGTGTCAAGACCTGCC-3′, and 5′-GTGGCATAGGTGGCAGACAT-3′; *Cd68*, 5′-ACCTGGACTACATGGCGGTG-3′ and 5′-AGAAGCTTTGGCCCAAGGGA-3′; adhesion Gprotein-coupled receptor E1 (*Adgre1*), 5′-GGCTTCCTGTCCAGCAATGG-3′ and 5′-GTGCAGACTGAGTTAGGACCAC-3′.

### Gene expression analyses using a public database

Comprehensive gene expression data of human tissues/cells was obtained from a public database, Functional Annotation of the Mammalian Genome 5 (FANTOM5) human promoterome (https://fantom.gsc.riken.jp/5/). Fragments per kilobase of exon per million mapped reads (FPKM) of genes in the tissues and cells were compared.

### Statistical analysis

Statistical analyses were performed with Prism 8 (GraphPad Software, USA). All the data were initially tested with *F* test or Bartlette’s test for normality distribution. If homoscedasticity could be assumed, they were analyzed with a parametric test using Student’s *t* test, one-way analysis of variance (ANOVA) followed by Dunnett’s multiple-comparison test. If homoscedasticity could not be assumed, Welch’s *t* test or Brown-Forsythe test followed by Dunnett’s T3 multiple-comparison test was applied. Differences with a *p* value of < 0.05 were considered significant: *p < 0.05, **p < 0.01, ***p < 0.001, and ****p < 0.0001; n. s., not significant. All data are expressed as the mean ± standard errors of the mean values.

## Results

### Macrophages attract mesenchymal cells via Emilin2

**Fig. 1:**
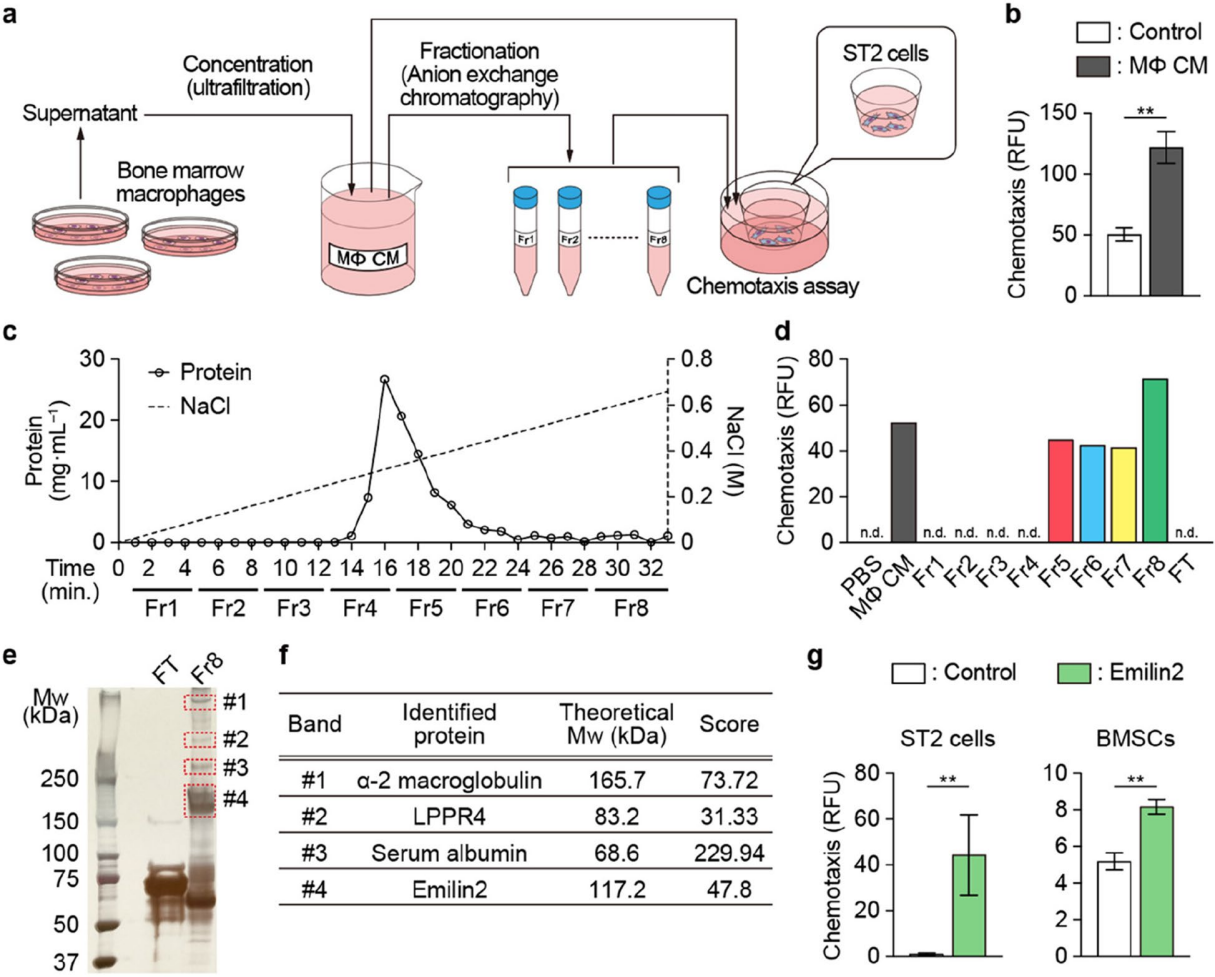
Emilin2 is a chemoattractant protein produced by macrophages that recruits mesenchymal cells. **a** Schematic diagram of the experiments. Conditioned medium of bone marrow macrophages underwent concentration and fractionation, and their chemoattractive activities were tested using ST2 cells. **b** Chemoattractive activity of the conditioned medium of bone marrow macrophages towards ST2 cells (*n* = 3). **c** Fractionation of conditioned medium of bone marrow macrophages. Protein concentration is indicated as a solid line, while the concentration of NaCl as a dotted line. **d** Chemoattractive activity of each fraction towards ST2 cells. **e** Image of silver staining of SDS-PAGE gel. Proteins in bands #1–4 (enclosed by red dotted lines) underwent Liquid chromatograph-tandem mass spectrometry (LC–MS/MS). **f** Proteins identified by LC–MS/MS, their theoretical molecular weight, and MASCOT scores are listed. **g** Chemoattractive activity of recombinant Emilin2 towards ST2 cells and bone marrow stromal cells (*n* = 4). Statistical analyses were carried out using Student’s *t* test or Welch’s *t* test. Error bars show the mean ± s.e.m. n.d., not detected. **p < 0.01. MΦ CM, macrophage-conditioned medium; Fr, fraction; FT, flow through; BMSC, bone marrow stromal cells

To identify macrophage-derived chemoattractants for mesenchymal cells, the migration assay was conducted using ST2 mesenchymal cells and the conditioned medium of bone marrow macrophages (Fig. 1a). The concentrated conditioned medium was shown to increase the chemotaxis of these mesenchymal cells (Fig. 1b). Then, the medium underwent fractionation by anion exchange chromatography, and the resultant fractions (Fr1 to 8) were examined to determine if they induce the migration of mesenchymal cells (Fig. 1a, c). Fr8 exhibited the highest chemoattractant capacity (Fig. 1d). The proteins contained in this fraction were identified by SDS-PAGE and liquid chromatography-tandem mass spectrometry (LC–MS/MS) (Fig. 1e, f, and Additional file 1: Fig. S1a, b). Among the identified proteins, only Emilin2 was a secreted protein [30, 31]. A chemotaxis assay conducted using recombinant Emilin2 protein confirmed that this protein induces the chemoattraction of mesenchymal cells (Fig. 1g).

Emilin2 was originally reported to be expressed in the lung and liver and was later found in other organs and tumors [31–33]. Using the public database, FANTOM5 human promoterome (https://fantom.gsc.riken.jp/5/), it was found that *EMILIN2* is highly expressed in organs that are abundant in macrophages (e.g., lung, lymph node, blood, and fat) (Additional file 2: Fig. S2a). A strong correlation was also found between the expression levels of *EMILIN2* and the macrophage marker genes, *CD14*, Fc fragment of IgG receptor 3a (*FCGR3A*), *CD68*, and integrin alpha M chain (*ITGAM*) (Additional file 2: Fig. S2b). In the bone, macrophages had higher expression of *Emilin2* than osteoclasts and osteoblasts (Additional file 2: Fig. S2c). These data suggest that macrophage-derived Emilin2 can exert its functions on bone metabolism via chemoattraction; however, there are few studies in the literature on its functions in chemoattraction and bone metabolism.

### Emilin2 deficiency results in impaired bone regeneration

To gain insights into the functions of Emilin2 in vivo, we generated *Emilin2*^–/–^ mice by introducing a deletion mutation, resulting in a frameshift to produce an abnormal peptide (Additional file 3: Fig. S3a–d). *Emilin2*^–/–^ mice were born with the expected Mendelian frequency, appeared grossly normal, grew normally, and were fertile (Additional file 3: Fig. S3e and data not shown). Micro-CT and histomorphometric analyses showed that *Emilin2*^–/–^ mice were osteopenic with decreased bone formation and increased bone resorption parameters (Additional file 3: Fig. S3f, g).

**Fig. 2:**
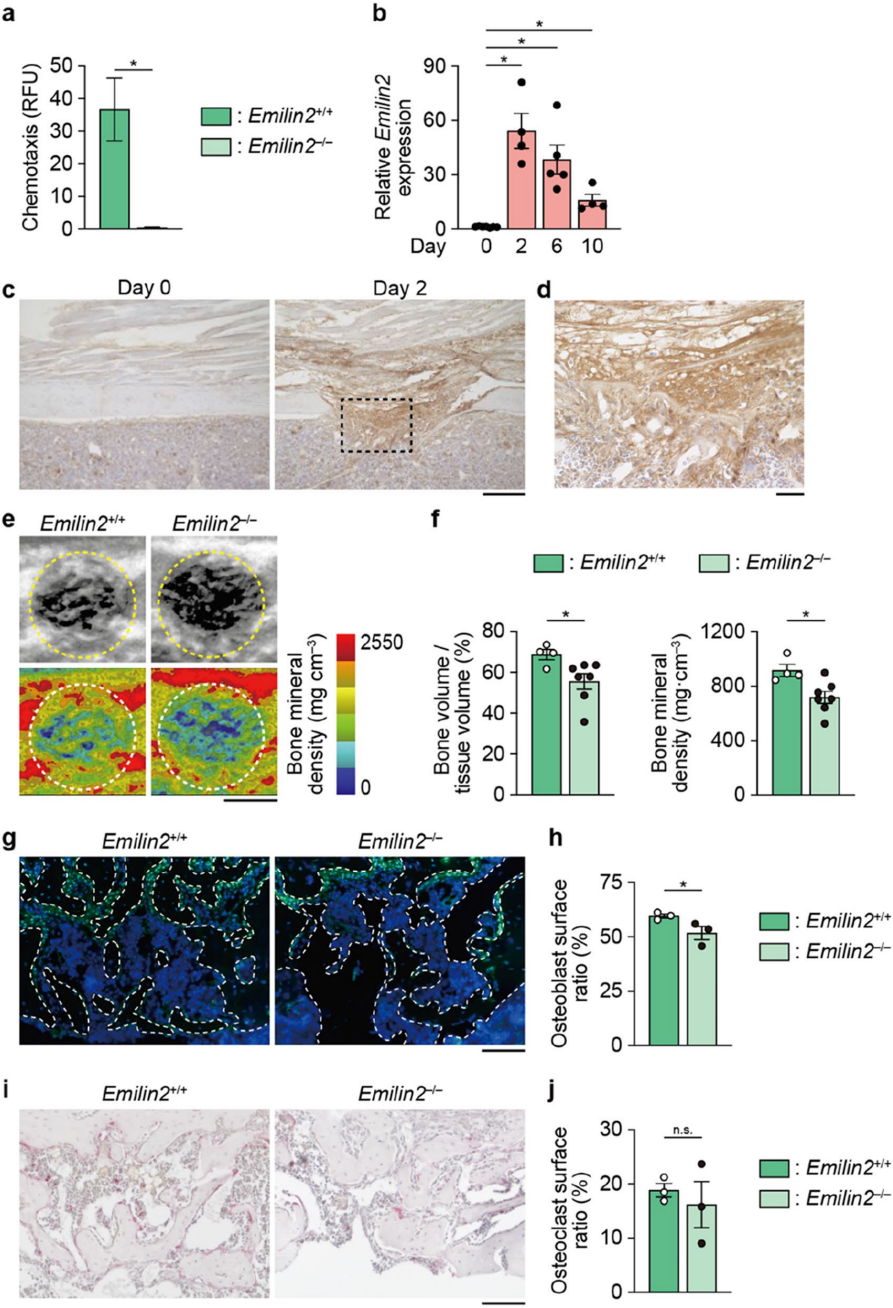
*Emilin2*^–/–^ mice exhibit delayed bone regeneration. **a** Chemoattractive activity of the macrophages harvested from *Emilin2*^+/+^ and *Emilin2*^–/–^ mice (*n* = 4). **b** Expression of *Emilin2* in the bone-surrounding tissue (*n* = 7, 4, 5, and 4, respectively). **c** Representative images of immunohistochemical analyses detecting Emilin2 (*n* = 3). Scale bar, 200 μm. **d** Magnified image of the area enclosed by dotted line in **c**. Scale bar, 40 μm. **e** Representative micro-computed tomography (CT) images (upper) and visualization of bone mineral density (BMD) (lower) of the drill hole in the *Emilin2*^+/+^ and *Emilin2*^–/–^ mice. Scale bar, 500 μm. **f** Quantification of newly formed bone in the drill hole and its BMD (*n* = 4 and 7, respectively). **g** Representative images of immunofluorescence detecting Osterix in the newly formed bone. Scale bar, 100 μm. **h** Quantification of Osterix^+^ osteoblast surface ratio (*n* = 3, 3 slides per mouse were analyzed). **i** Representative images of tartrate-resistant acid phosphatase (TRAP) staining of the newly formed bone. Scale bar, 100 μm. **j** Quantification of TRAP^+^ osteoclast surface ratio (*n* = 3, 3 slides per mouse were analyzed). For the comparison of the 2 groups, statistical analyses were carried out using Student’s *t* test or Welch’s *t* test. For the multiple comparisons, Brown-Forsythe ANOVA test followed by Dunnett’s T3 test was carried out. Error bars show the mean ± s.e.m. *p < 0.05; n.s., not significant

Using macrophages obtained from the *Emilin2*^–/–^ mice, a chemotaxis assay was conducted. The results showed that *Emilin2* deficiency severely impaired the chemoattractant capacity of macrophages towards mesenchymal cells (Fig. 2a), indicating that Emilin2 is the major chemoattractant of macrophages that recruits mesenchymal cells. *EMILIN2* expression was correlated with M1 macrophage markers but not M2, and its expression was upregulated by microbial infection (Additional file 4: Fig. S4a, b), suggesting a link between EMILIN2 and acute inflammation.

Because the process of bone regeneration after fracture involves both macrophages and mesenchymal cells [4], we hypothesized that Emilin2 produced by macrophages plays a role in attracting mesenchymal cells to promote bone regeneration. To test this hypothesis, the bone regeneration model was adopted because both the inflammation and regeneration processes take place [6]. *Emilin2* expression was robustly induced in the surrounding tissue of the bone injury site (Fig. 2b), but not in the bone marrow (Additional file 4: Fig. S4c). Emilin2 was found to be deposited in the clot formed in the bony defect (Fig. 2c,d), suggesting that Emilin2 recruits mesenchymal cells to the clot, the initiation site of bone regeneration. Micro-CT analysis revealed that there was an impairment in bone regeneration in *Emilin2*^–/–^ mice (Fig. 2e, f). The number of osteoblasts, but not osteoclasts, was decreased in these mice (Fig. 2g–j).

### Emilin2 recruits mesenchymal cells to the site of bone injury

**Fig. 3:**
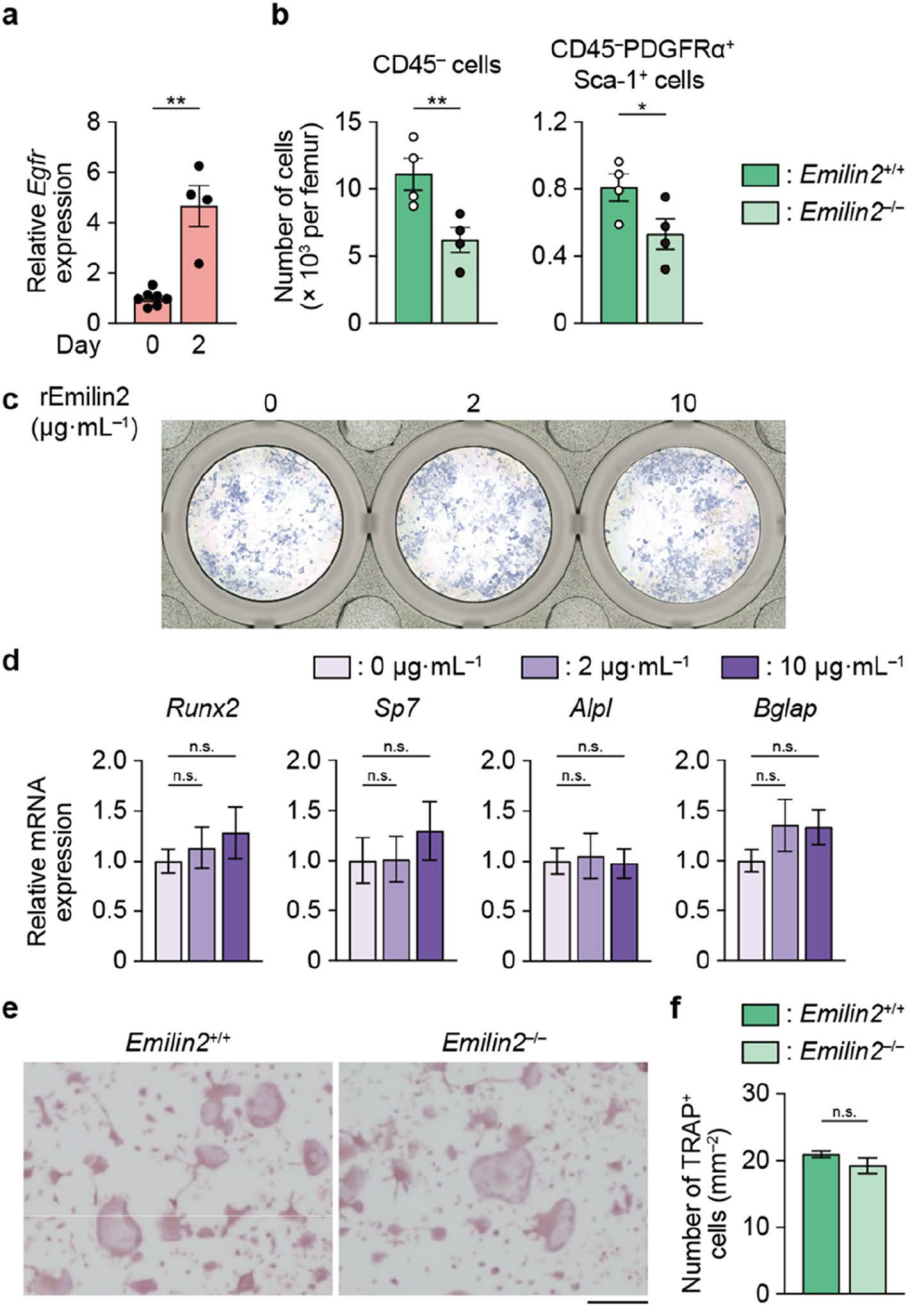
Emilin2 promotes the migration of mesenchymal cells into the injury tissue. **a** mRNA expression of *Egfr* in bone-surrounding tissue (*n* = 7 and 4, respectively). **b** The number of mesenchymal cells in the bone injury tissue of *Emilin2*^+/+^ and *Emilin2*^–/–^ mice (*n* = 4). **c** Representative alkaline phosphatase (ALP) staining images of osteoblasts. **d** mRNA expression of osteogenic genes. These experiments were repeated 3 times with replicates of 3 wells. **e** Representative images of tartrate-resistant acid phosphatase (TRAP) staining of osteoclasts generated from bone marrow macrophages of *Emilin2*^+/+^ and *Emilin2*^–/–^ mice. Scale bar, 500 μm. **f** Quantification of TRAP^+^ osteoclasts (triplicate of 3 biological replicates). For the comparison of the 2 groups, statistical analyses were carried out using Student’s *t* test or Welch’s *t* test. For multiple comparisons, one-way ANOVA followed by Dunnett’s multiple-comparison test was conducted. Error bars show the mean ± s.e.m. *p < 0.05; **p < 0.01; n.s., not significant

The decreased osteoblast number at the injury site of *Emilin2*^–/–^ mice could be due to impairment of the chemoattraction of mesenchymal progenitor cells for osteoblasts and/or the impairment of osteoblastic differentiation of these cells. It was shown after injury that there was increased expression of the Emilin2 receptor, *Egfr *[34], in the tissue surrounding the bone but not in the bone marrow (Fig. 3a and Additional file 4: Fig. S4d). Thus, mesenchymal cells that appeared after injury underwent further investigation. Compared to *Emilin2*^+/+^ mice, there was a smaller number of CD45^–^ mesenchymal cells, including platelet-derived growth factor receptor *α* (PDGFRα)^+^ stem cells antigen-1 (Sca-1)^+^ cells (PαS cells), in the injured tissue of *Emilin2*^–/–^ mice (Fig. 3b). By contrast, the number of CD11b^+^ cells was similar in *Emilin2*^–/–^ mice compared to *Emilin2*^+/+^ mice (Additional file 4: Fig. S4e).

Next, the role of Emilin2 in osteoblastogenesis was assessed. The addition of recombinant Emilin2 did not result in the obvious progression of osteoblastogenesis of the cells harvested from the injury site (Fig. 3c, d), although Emilin2 deficiency resulted in decreased bone formation in the process of bone regeneration (Fig. 2g, h). These data indicate that Emilin2 produced after bone injury attracts mesenchymal cells but does not promote osteoblastogenesis. Osteoclastogenesis of *Emilin2*^–/–^ bone marrow cells exhibited no significant difference compared to *Emilin2*^+/+^ cells (Fig. 3e, f), consistent with the results observed at the bone regeneration site (Fig. 2i, j).

### Emilin2 in the bone-surrounding tissue enhances bone regeneration

**Fig. 4:**
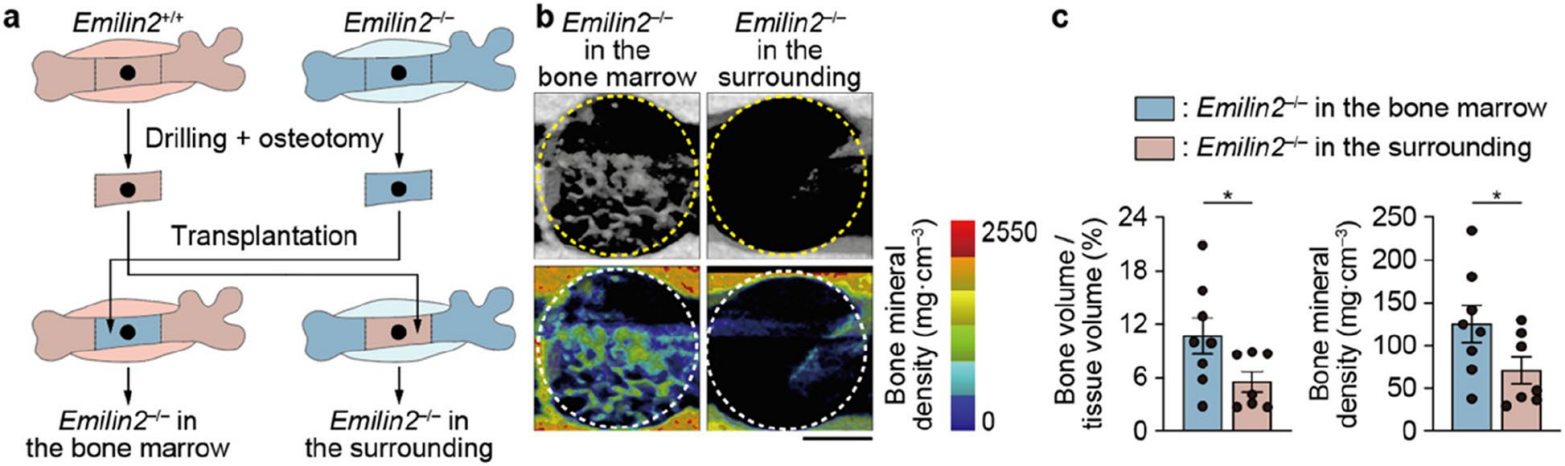
Emilin2 from the surrounding tissue of the injured bone promotes bone regeneration. **a** Schematic diagram of the transplantation experiment. **b** Representative micro-computed tomography (CT) images (upper) and visualization of bone mineral density (BMD) (lower) of the drill hole in mice deficient in *Emilin2* in the bone marrow or in the surrounding tissue of the bone. Scale bar, 500 μm. **c** Quantification of newly formed bone in the drill hole and its BMD (*n* = 8 and 7, respectively). Statistical analyses were carried out using Student’s *t* test. Error bars show the mean ± s.e.m. *p < 0.05

Coverage of fractured bone by the surrounding soft tissue (i.e., muscle and fasciocutaneous flaps) is crucial for bone regeneration. These tissues are thought to function as a pool of skeletal stem cells, immune cells, and cytokines that regulate inflammation, vascularization, and cell differentiation [35, 36]. Because Emilin2 expression is upregulated in the homogenized bone-surrounding tissue (i.e., periosteum; muscle, including fascia and interstitium; and granulation tissue after injury) but not in the bone marrow after injury (Fig. 2b and Additional file 4: Fig. S4c), it has been suggested that Emilin2 in the bone-surrounding tissue enhances bone regeneration. To address this hypothesis, we used mice deficient in Emilin2 in either the bone-surrounding tissue or the bone marrow. A pair of *Emilin2*^+/+^ and *Emilin2*^–/–^ mice underwent drill-hole injury, after which the diaphyses of these mice were resected and transplanted into their counterparts (Fig. 4a). Mice with Emilin2 deficiency in the bone-surrounding tissue exhibited a lower level of bone regeneration compared to their counterparts (Fig. 4b, c). These data indicate that Emilin2 in the bone-surrounding tissue facilitates bone regeneration.

### Administration of Emilin2 enhances bone regeneration

**Fig. 5:**
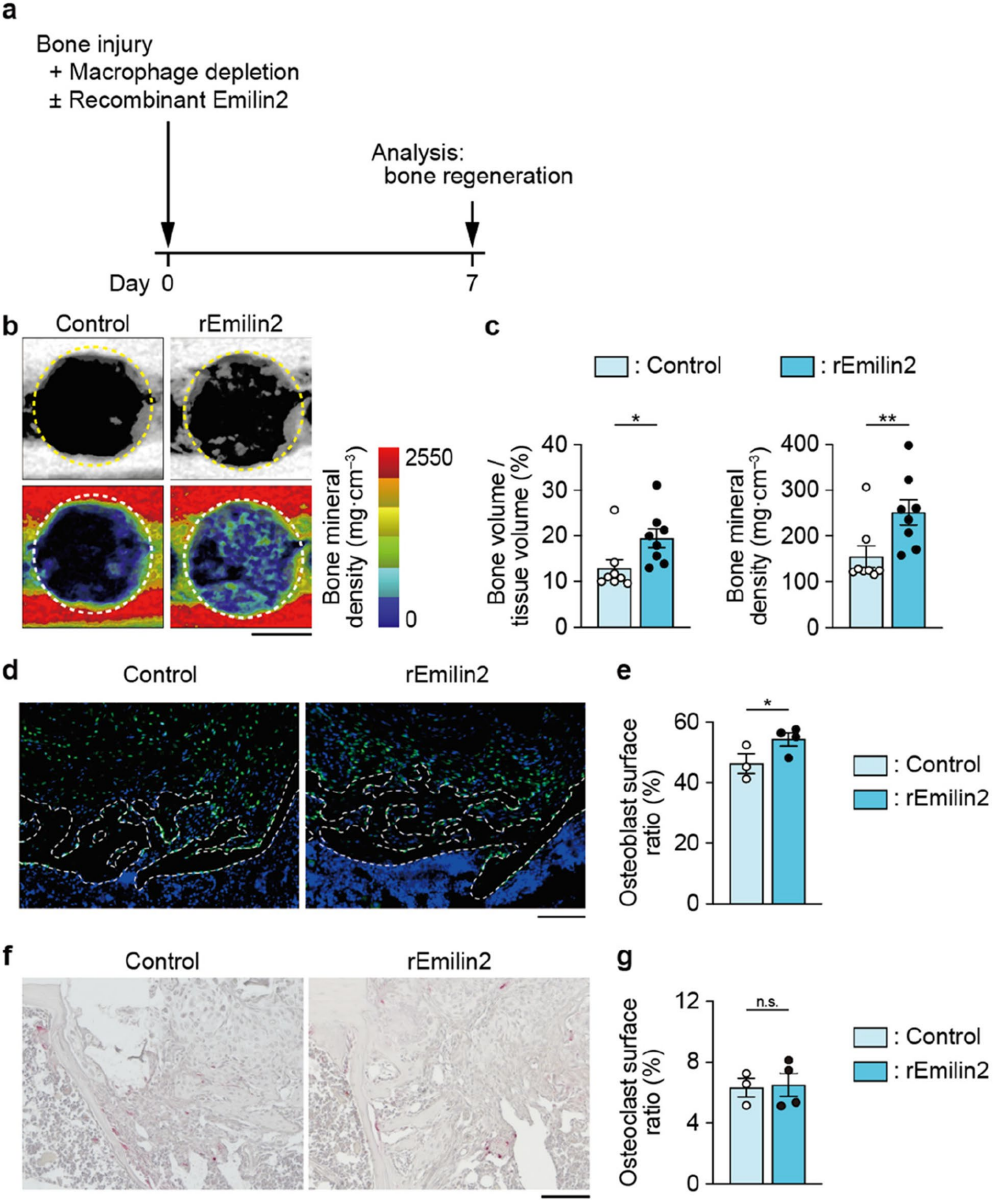
Emilin2 treatment promotes bone regeneration in the absence of macrophages. **a** Schematic diagram of the treatment of bone injury by Emilin2. **b** Representative micro-computed tomography (CT) images (upper) and visualization of bone mineral density (BMD) (lower) of the drill hole in mice treated with or without Emilin2. Scale bar, 500 μm. **c** Quantification of newly formed bone in the drill hole and its BMD (*n* = 8). **d** Representative images of immunofluorescence detecting Osterix in the newly formed bone. Scale bar, 100 μm. **e** Quantification of Osterix^+^ osteoblast surface ratio (*n* = 3 and 4, 3–5 slides per mouse were analyzed). **f** Representative images of tartrate-resistant acid phosphatase (TRAP) staining of the newly formed bone. Scale bar, 100 μm. **g** Quantification of TRAP^+^ osteoclast surface ratio (*n* = 3 and 4, 3 slides per mouse were analyzed). Statistical analyses were carried out using Student’s *t* test. Error bars show the mean ± s.e.m. *p < 0.05; **p < 0.01

Finally, in light of the clinical application of Emilin2 for the treatment of bone fracture, recombinant Emilin2 was introduced to the injury site using a collagen carrier, at the same time as macrophage depletion in the bone-surrounding tissue (Fig. 5a and Additional file 5: Fig. S5a, b). Micro-CT analysis showed that Emilin2 administration significantly accelerated bone regeneration (Fig. 5b, c). Histologically, a significantly large number of osteoblasts was observed at the bone injury site upon the addition of recombinant Emilin2 (Fig. 5d, e). By contrast, the number of osteoclasts was comparable in Emilin2-treated and untreated mice (Fig. 5f, g).

Thus, our results demonstrated that Emilin2 is produced after bone injury and deposited at the injury site. Macrophages produce Emilin2 to facilitate the migration of mesenchymal cells. The recruitment of mesenchymal cells results in bone regeneration. The results suggest that local administration of Emilin2 may be a novel option for treating bone fracture.

## Discussion

Bone fracture is one of the most debilitating conditions, as patients suffer from pain, immobility, bone deformity, and nonunion. The increased aging of the worldwide population has led to a substantial increase in the incidence and prevalence of bone fracture, and years lived with disability. Therefore, costs for clinical and social care have become a serious burden not only to patients but also to their families and society [37]. Prolonged fixation of the fractured bone significantly impairs physical activity and can result in being bedridden. Thus, there is a great demand to establish treatment strategies that can accelerate the regenerative process of fractured bone.

Bone fracture is generally treated by closed or open reduction and fixation. If a bony defect is excessively large, bone grafting will be added to the procedure. Drugs targeting bone metabolism (teriparatide, vitamin D, and calcium), as well as antibiotics and analgesics are also employed to treat fractures [38]. The immune system has recently emerged as a promising target for bone fracture healing, and some clinical trials are underway. Because the inflammatory milieu is necessary for bone regeneration, it is crucial that immunotherapies do not disrupt the inflammatory milieu.

Mesenchymal stem/progenitor cells possess a high potential of tissue regeneration by proliferating and differentiating into cells constituting the tissue. Although tremendous efforts are being made by researchers and clinicians, there have been no established stem cell therapies for bone fracture to date, possibly due to difficulties in manipulating these cells [5, 12, 13]. We hypothesized that the mobilization of endogenous stem cells would be another mode of stem cell therapy. Based on this idea, Emilin2 was discovered to be a chemoattractant for mesenchymal progenitor cells, coupling bone injury and regeneration processes (Additional file 1: Fig. S1a–g, 2a).

Emilin2 is a glycoprotein that belongs to the Emilin/Multimerin family. Emilin1 and Emilin2 have high homology and are both abundant in the ECM, establishing ECM-ECM connections between matrix molecules including elastin and fibrillin [30]. Emilin proteins in the ECM in organs play roles in the development and functions of various organs including the heart, skin, digit, and inner ear [33, 39–41]. Emilin proteins can facilitate ECM-cell adhesion as well. Mesenchymal cells including fibroblasts reportedly adhere to Emilin1, and it has been speculated that Emilin2 also attracts cells expressing its receptors [32, 42].

In this study, Emilin2 was detected after bone injury and accumulated in the clot (Fig. 2c, d), consistent with previous reports describing the deposition of Emilin2 in the thrombus, a form of blood clot [43, 44]. The molecular weight of Emilin2 exceeds 100 kDa (Additional file 1: Fig. S1b), which is far larger than that of cytokines and chemokines. Therefore, Emilin2 at the bone injury site functions as a mark of migration of mesenchymal cells deposited at the site of injury (i.e., clot), rather than entering the circulation. Mesenchymal cells accumulated at the injury site promote bone regeneration by differentiating into osteoblasts and forming new bone tissue. Local administration of Emilin2 to the site of bone injury successfully improved bone regeneration (Fig. 5a–c), suggesting that it recruits endogenous stem/progenitor cells for tissue regeneration.

## Conclusions

We demonstrated that macrophages produce Emilin2. Emilin2 at the bone injury site recruits mesenchymal progenitor cells to the injury site, enhancing bone regeneration. Thus, local administration of Emilin2 promotes bone regeneration, suggesting its clinical application in treating bone fractures.

### Supplementary Information

Additional file 1: Fig. S1. Emilin2 is detected in the fraction of conditioned medium of macrophages with chemoattractive activity towards mesenchymal cells. a Amino acid sequence of murine Emilin2 protein. Peptide fragments highlighted in red were detected in fraction 8 by LC-MS/MS analysis (see Fig. 1e, f). b Western blotting analysis of Emilin2 in the macrophage-conditioned medium (MΦ CM).

Additional file 2: Fig. S2. Emilin2 is expressed highly in macrophages. a Comprehensive analysis of *EMILIN2* expression in human organs. b The correlation between *EMILIN2* and macrophage marker genes in human cells. Data was obtained from public databases, FANTOM5 human promoterome (https://fantom.gsc.riken.jp/5/). c mRNA expression of *Emilin2* in mouse cells. For the multiple comparisons, Brown-Forsythe ANOVA test followed by Dunnett’s T3 test was carried out. Error bars show the mean ± s.e.m. *p < 0.05. FPKM: fragments per kilobase of exon per million reads mapped.

Additional file 3: Fig. S3. Generation of *Emilin2*^–/–^ mice. a Deletion of a single nucleotide in the *Emilin2* gene. Adenine at 24 base pairs downstream from the first ATG was deleted from the genomic DNA. b PCR analysis for genotyping. c Emilin2 translation products of *Emilin2*^+/+^ and *Emilin2*^–/–^ mice. Different amino acids and stop codons are highlighted in red. *: stop codon. d Western blotting and coomassie brilliant blue (CBB) staining images of the conditioned medium of *Emilin2*^+/+^ or *Emilin2*^–/–^ macrophages. e Body weight of *Emilin2*^+/+^ and *Emilin2*^–/–^ mice. f Representative 3D images of cancellous bone of proximal tibia, reconstructed from micro-CT images. Scale bar, 1 mm. g Bone parameters obtained by micro-CT analyses and histomorphometric analyses (*n* = 8). Statistical analyses were carried out using Student’s *t* test or Welch’s *t* test. Error bars show the mean ± s.e.m. *p < 0.05; **p < 0.01; n.s., not significant.

Additional file 4:. Fig. S4. Emilin2 is expressed upon acute inflammation. a The correlation between EMILIN2 and M1/M2 macrophage marker genes in human cells. b *EMILIN2* expression by CD14^+^ human monocytes upon stimulation. Data was obtained from public databases, FANTOM5 human promoterome (https://fantom.gsc.riken.jp/5/). c mRNA expression of *Emilin2* in mouse bone marrow after bone injury. d mRNA expression of *Egfr* in mouse bone marrow after bone injury. e The number of CD11b^+^ cells in the bone injury tissue of *Emilin2*^+/+^ and *Emilin2*^–/–^ mice (*n* = 4). For the comparison of the 2 groups, statistical analyses were carried out using Student’s *t* test or Welch’s *t* test. For the multiple comparison, one-way ANOVA test followed by Dunnett’s test was carried out. Error bars show the mean ± s.e.m. n.s., not significant. FPKM: fragments per kilobase of exon per million reads mapped.

Additional file 5: Fig. S5. Local administration of clodronate liposome reduces macrophages in the bone-surrounding tissue but not in the bone marrow. a mRNA expression of macrophage marker genes in the bone-surrounding tissue. b mRNA expression of macrophage marker genes and *Emilin2* in the bone marrow (*n* = 4 and 5, respectively). Statistical analyses were carried out using Student’s *t* test or Welch’s *t* test. Error bars show the mean ± s.e.m. *p < 0.05; ***p < 0.001; ****p < 0.0001; n.s., not significant. MΦ depletion: macrophage depletion.

## Data Availability

The datasets used and/or analyzed during the current study are available from the corresponding author on reasonable request.

## Abbreviations

ECM: Extracellular matrix
Emilin: Elastin microfibril interface located protein
Emilin1: Elastin microfibril interface located protein 1
Emilin2: Elastin microfibril interface located protein 2
Gapdh: Glyceraldehyde-3-phosphate dehydrogenase
Bglap: Bone γ-carboxyglutamate protein
Alpl: Alkaline phosphatase
Sp7: Sp7 transcription factor
Runx2: Runt-related transcription factor 2
Adgre1: Adhesion G-protein-coupled receptor E1
CXCL12: C–X–C motif chemokine 12
CXCR4: C–X–C chemokine receptor type 4
SDS-PAGE: Sodium dodecyl sulfate–polyacrylamide gel electrophoresis
LC–MS/MS: Liquid chromatography-tandem mass spectrometry
CD8α: Cluster of differentiation 8α
CD11b: Cluster of differentiation 11b
CD14: Cluster of differentiation 14
CD16/32: Cluster of differentiation 16/32
CD31: Cluster of differentiation 31
CD45: Cluster of differentiation 45
CD68: Cluster of differentiation 68
CD140α: Cluster of differentiation 140α
FCGR3A: Fc fragment of IgG receptor 3a
ITGAM: Integrin alpha M chain
Micro-CT: Microcomputed tomography
Egfr: Epidermal growth factor receptor
Sca-1: Stem cell antigen-1
PDGFRα: Platelet-derived growth factor receptor α
PαS cells: PDGFRα^+^Sca-1^+^ cells
PE: Phycoerythrin
PBS: Phosphate-buffered saline
PFA: Paraformaldehyde
GMA: Glycol methacrylate
EDTA: Ethylenediaminetetraacetic acid
DAB: Diaminobenzidine
7-AAD: 7-Amino-actinomycin D
M-CSF: Macrophage colony-stimulating factor
LC: Liquid chromatography
MACS: Magnetic-activated cell sorting
FBS: Fetal bovine serum
MEM: Minimum essential medium
RANKL: Receptor activator of nuclear factor kappa-Β ligand
TRAP: Tartrate-resistant acid phosphatase
ALP: Alkaline phosphatase
qRT-PCR: Quantitative reverse transcriptase-polymerase chain reaction
FPKM: Fragments per kilobase of exon per million mapped reads
BMD: Bone mineral density
FANTOM5: Functional Annotation of the Mammalian Genome 5
CBB: Coomassie brilliant blue
ANOVA: One-way analysis of variance
NCGG: National Center for Geriatrics and Gerontology

## References

[1] Kim JY, Ohn J, Yoon JS, Kang BM, Park M, Kim S (2019). Priming mobilization of hair follicle stem cells triggers permanent loss of regeneration after alkylating chemotherapy. Nat Commun.

[2] Murphy MP, Koepke LS, Lopez MT, Tong X, Ambrosi TH, Gulati GS (2020). Articular cartilage regeneration by activated skeletal stem cells. Nat Med.

[3] Baak LM, Wagenaar N, van der Aa NE, Groenendaal F, Dudink J, Tataranno ML (2022). Feasibility and safety of intranasally administered mesenchymal stromal cells after perinatal arterial ischaemic stroke in the Netherlands (PASSIoN): a first-in-human, open-label intervention study. Lancet Neurol.

[4] Ono T, Takayanagi H (2017). Osteoimmunology in bone fracture healing. Curr Osteoporos Rep.

[5] Duda GN, Geissler S, Checa S, Tsitsilonis S, Petersen A, Schmidt-Bleek K (2023). The decisive early phase of bone regeneration. Nat Rev Rheumatol.

[6] Ono T, Okamoto K, Nakashima T, Nitta T, Hori S, Iwakura Y (2016). IL-17-producing gammadelta T cells enhance bone regeneration. Nat Commun.

[7] Matsushita Y, Nagata M, Kozloff KM, Welch JD, Mizuhashi K, Tokavanich N (2020). A Wnt-mediated transformation of the bone marrow stromal cell identity orchestrates skeletal regeneration. Nat Commun.

[8] Julien A, Kanagalingam A, Martinez-Sarra E, Megret J, Luka M, Menager M (2021). Direct contribution of skeletal muscle mesenchymal progenitors to bone repair. Nat Commun.

[9] Jeffery EC, Mann TLA, Pool JA, Zhao Z, Morrison SJ (2022). Bone marrow and periosteal skeletal stem/progenitor cells make distinct contributions to bone maintenance and repair. Cell Stem Cell.

[10] Ambrosi TH, Marecic O, McArdle A, Sinha R, Gulati GS, Tong X (2021). Aged skeletal stem cells generate an inflammatory degenerative niche. Nature.

[11] Cowan CM, Shi YY, Aalami OO, Chou YF, Mari C, Thomas R (2004). Adipose-derived adult stromal cells heal critical-size mouse calvarial defects. Nat Biotechnol.

[12] Thurairajah K, Briggs GD, Balogh ZJ (2021). Stem cell therapy for fracture non-union: the current evidence from human studies. J Orthop Surg.

[13] Zhang K, Cheng K (2023). Stem cell-derived exosome versus stem cell therapy. Nat Rev Bioeng.

[14] Kitaori T, Ito H, Schwarz EM, Tsutsumi R, Yoshitomi H, Oishi S (2009). Stromal cell-derived factor 1/CXCR4 signaling is critical for the recruitment of mesenchymal stem cells to the fracture site during skeletal repair in a mouse model. Arthritis Rheum.

[15] Ho CY, Sanghani A, Hua J, Coathup M, Kalia P, Blunn G (2015). Mesenchymal stem cells with increased stromal cell-derived factor 1 expression enhanced fracture healing. Tissue Eng Part A.

[16] Glass GE, Chan JK, Freidin A, Feldmann M, Horwood NJ, Nanchahal J (2011). TNF-alpha promotes fracture repair by augmenting the recruitment and differentiation of muscle-derived stromal cells. Proc Natl Acad Sci U S A.

[17] Varol C, Mildner A, Jung S (2015). Macrophages: development and tissue specialization. Annu Rev Immunol.

[18] Vi L, Baht GS, Soderblom EJ, Whetstone H, Wei Q, Furman B (2018). Macrophage cells secrete factors including LRP1 that orchestrate the rejuvenation of bone repair in mice. Nat Commun.

[19] Yahara Y, Barrientos T, Tang YJ, Puviindran V, Nadesan P, Zhang H (2020). Erythromyeloid progenitors give rise to a population of osteoclasts that contribute to bone homeostasis and repair. Nat Cell Biol.

[20] Ono T, Nakashima T (2022). Oral bone biology. J Oral Biosci.

[21] Kohara Y, Kitazawa R, Haraguchi R, Imai Y, Kitazawa S (2022). Macrophages are requisite for angiogenesis of type H vessels during bone regeneration in mice. Bone.

[22] Nagashima M, Sakai A, Uchida S, Tanaka S, Tanaka M, Nakamura T (2005). Bisphosphonate (YM529) delays the repair of cortical bone defect after drill-hole injury by reducing terminal differentiation of osteoblasts in the mouse femur. Bone.

[23] Niwa H, Yamamura K, Miyazaki J (1991). Efficient selection for high-expression transfectants with a novel eukaryotic vector. Gene.

[24] Takeshita S, Fumoto T, Matsuoka K, Park KA, Aburatani H, Kato S (2013). Osteoclast-secreted CTHRC1 in the coupling of bone resorption to formation. J Clin Invest.

[25] Bouxsein ML, Boyd SK, Christiansen BA, Guldberg RE, Jepsen KJ, Muller R (2010). Guidelines for assessment of bone microstructure in rodents using micro-computed tomography. J Bone Miner Res.

[26] Dempster DW, Compston JE, Drezner MK, Glorieux FH, Kanis JA, Malluche H (2013). Standardized nomenclature, symbols, and units for bone histomorphometry: a 2012 update of the report of the ASBMR Histomorphometry Nomenclature Committee. J Bone Miner Res.

[27] Takeshita S, Kaji K, Kudo A (2000). Identification and characterization of the new osteoclast progenitor with macrophage phenotypes being able to differentiate into mature osteoclasts. J Bone Miner Res.

[28] Kim Y, Hayashi M, Ono T, Yoda T, Takayanagi H, Nakashima T (2020). Suppression of hematopoietic cell kinase ameliorates the bone destruction associated with inflammation. Mod Rheumatol.

[29] Tsuchiya Y, Hayashi M, Nagamatsu K, Ono T, Kamakura M, Iwata T (2020). The key royal jelly component 10-hydroxy-2-decenoic acid protects against bone loss by inhibiting NF-kappaB signaling downstream of FFAR4. J Biol Chem.

[30] Colombatti A, Doliana R, Bot S, Canton A, Mongiat M, Mungiguerra G (2000). The EMILIN protein family. Matrix Biol.

[31] Doliana R, Bot S, Mungiguerra G, Canton A, Cilli SP, Colombatti A (2001). Isolation and characterization of EMILIN-2, a new component of the growing EMILINs family and a member of the EMI domain-containing superfamily. J Biol Chem.

[32] Colombatti A, Spessotto P, Doliana R, Mongiat M, Bressan GM, Esposito G (2011). The EMILIN/Multimerin family. Front Immunol.

[33] Russell IJ, Lukashkina VA, Levic S, Cho Y-W, Lukashkin AN, Ng L (2020). Emilin 2 promotes the mechanical gradient of the cochlear basilar membrane and resolution of frequencies in sound. Sci Adv.

[34] Paulitti A, Andreuzzi E, Bizzotto D, Pellicani R, Tarticchio G, Marastoni S (2018). The ablation of the matricellular protein EMILIN2 causes defective vascularization due to impaired EGFR-dependent IL-8 production affecting tumor growth. Oncogene.

[35] Harry LE, Sandison A, Paleolog EM, Hansen U, Pearse MF, Nanchahal J (2008). Comparison of the healing of open tibial fractures covered with either muscle or fasciocutaneous tissue in a murine model. J Orthop Res.

[36] Chan JK, Harry L, Williams G, Nanchahal J (2012). Soft-tissue reconstruction of open fractures of the lower limb: muscle versus fasciocutaneous flaps. Plast Reconstr Surg.

[37] Collaborators GBDF (2021). Global, regional, and national burden of bone fractures in 204 countries and territories, 1990–2019: a systematic analysis from the Global Burden of Disease Study 2019. Lancet Healthy Longev.

[38] Gomberg SJ, Wustrack RL, Napoli N, Arnaud CD, Black DM (2011). Teriparatide, vitamin D, and calcium healed bilateral subtrochanteric stress fractures in a postmenopausal woman with a 13-year history of continuous alendronate therapy. J Clin Endocrinol Metab.

[39] Schiavinato A, Keene DR, Wohl AP, Corallo D, Colombatti A, Wagener R (2016). Targeting of EMILIN-1 and EMILIN-2 to fibrillin microfibrils facilitates their incorporation into the extracellular matrix. J Invest Dermatol.

[40] Hurle JM, Kitten GT, Sakai LY, Volpin D, Solursh M (1994). Elastic extracellular matrix of the embryonic chick heart: an immunohistological study using laser confocal microscopy. Dev Dyn.

[41] Hurle JM, Colombatti A (1996). Extracellular matrix modifications in the interdigital spaces of the chick embryo leg bud during the formation of ectopic digits. Anat Embryol.

[42] Spessotto P, Cervi M, Mucignat MT, Mungiguerra G, Sartoretto I, Doliana R (2003). beta 1 Integrin-dependent cell adhesion to EMILIN-1 is mediated by the gC1q domain. J Biol Chem.

[43] Sa Q, Hoover-Plow JL (2011). EMILIN2 (Elastin microfibril interface located protein), potential modifier of thrombosis. Thromb J.

[44] Huang M, Sannaningaiah D, Zhao N, Gong Y, Grondolsky J, Hoover-Plow J (2015). EMILIN2 regulates platelet activation, thrombus formation, and clot retraction. PLoS ONE.

