# Supplementary material for: Emilin2 marks the target region for mesenchymal cell accumulation in bone regeneration
Source: Inflamm Regen. 2024 Jun 3;44:27. doi: 10.1186/s41232-024-00341-6 (PMC11145771; doi:10.1186/s41232-024-00341-6)
Supplement: Supplementary file 1 — Additional file 1: Fig. S1. Emilin2 is detected in the fraction of conditioned medium of macrophages with chemoattractive activity towards mesenchymal cells. a Amino acid sequence of murine Emilin2 protein. Peptide fragments highlighted in red were detected in fraction 8 by LC-MS/MS analysis (see Fig. 1e, f). b Western blotting analysis of Emilin2 in the macrophage-conditioned medium (MΦ CM). [file 41232_2024_341_MOESM1_ESM.docx]

**
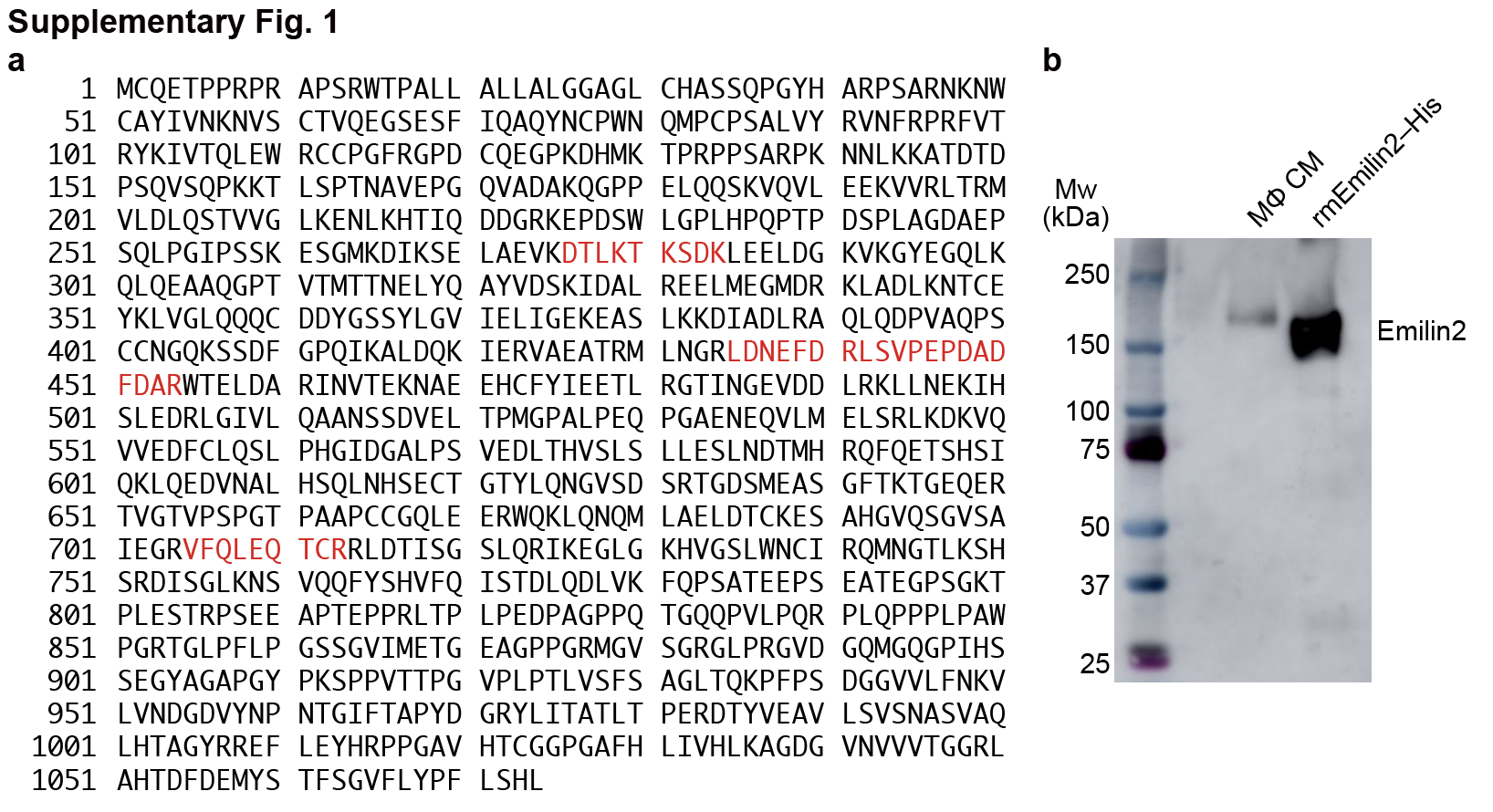
Supplementary figure legends**

**Supplementary Fig. 1** Emilin2 is detected in the fraction of conditioned medium of macrophages with chemoattractive activity towards mesenchymal cells. **a** Amino acid sequence of murine Emilin2 protein. Peptide fragments highlighted in red were detected in the fraction 8 by LC-MS/MS analysis (see **Fig. 1e, f**). **b** Western blotting analysis of Emilin2 in the macrophage–conditioned medium (MΦ CM).
