# Supplementary material for: Emilin2 marks the target region for mesenchymal cell accumulation in bone regeneration
Source: Inflamm Regen. 2024 Jun 3;44:27. doi: 10.1186/s41232-024-00341-6 (PMC11145771; doi:10.1186/s41232-024-00341-6)
Supplement: Supplementary file 2 — Additional file 2: Fig. S2. Emilin2 is expressed highly in macrophages. a Comprehensive analysis of EMILIN2 expression in human organs. b The correlation between EMILIN2 and macrophage marker genes in human cells. Data was obtained from public databases, FANTOM5 human promoterome (https://fantom.gsc.riken.jp/5/). c mRNA expression of Emilin2 in mouse cells. For the multiple comparisons, Brown-Forsythe ANOVA test followed by Dunnett’s T3 test was carried out. Error bars show the mean ± s.e.m. *p < 0.05. FPKM: fragments per kilobase of exon per million reads mapped. [file 41232_2024_341_MOESM2_ESM.docx]

**
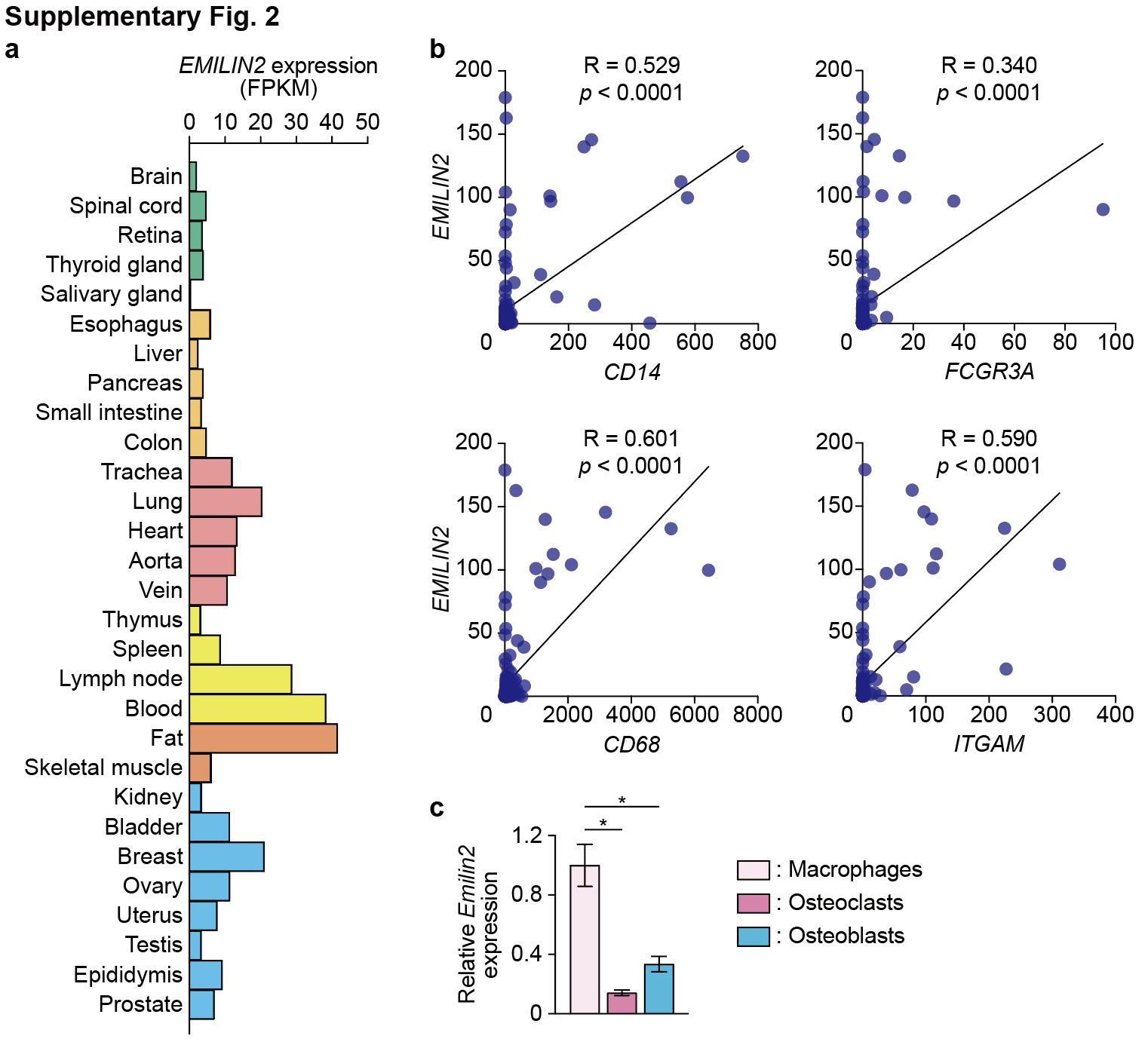
Supplementary Fig. 2** Emilin2 is expressed highly in macrophages. **a** Comprehensive analysis of *EMILIN2* expression in human organs. **b** The correlation between *EMILIN2* and macrophage marker genes in human cells. Data was obtained from public databases, FANTOM5 human promoterome (https://fantom.gsc.riken.jp/5/). **c** mRNA expression of *Emilin2* in mouse cells. For the multiple comparison, Brown–Forsythe ANOVA test followed by Dunnett’s T3 test was carried out. Error bars show the mean ± s.e.m. **p* < 0.05. FPKM: fragments per kilobase of exon per million reads mapped.
