# Supplementary material for: Emilin2 marks the target region for mesenchymal cell accumulation in bone regeneration
Source: Inflamm Regen. 2024 Jun 3;44:27. doi: 10.1186/s41232-024-00341-6 (PMC11145771; doi:10.1186/s41232-024-00341-6)
Supplement: Supplementary file 3 — Additional file 3: Fig. S3. Generation of Emilin2–/– mice. a Deletion of a single nucleotide in the Emilin2 gene. Adenine at 24 base pairs downstream from the first ATG was deleted from the genomic DNA. b PCR analysis for genotyping. c Emilin2 translation products of Emilin2+/+ and Emilin2–/– mice. Different amino acids and stop codons are highlighted in red. *: stop codon. d Western blotting and coomassie brilliant blue (CBB) staining images of the conditioned medium of Emilin2+/+ or Emilin2–/– macrophages. e Body weight of Emilin2+/+ and Emilin2–/– mice. f Representative 3D images of cancellous bone of proximal tibia, reconstructed from micro-CT images. Scale bar, 1 mm. g Bone parameters obtained by micro-CT analyses and histomorphometric analyses (n = 8). Statistical analyses were carried out using Student’s t test or Welch’s t test. Error bars show the mean ± s.e.m. *p < 0.05; **p < 0.01; n.s., not significant. [file 41232_2024_341_MOESM3_ESM.docx]

**
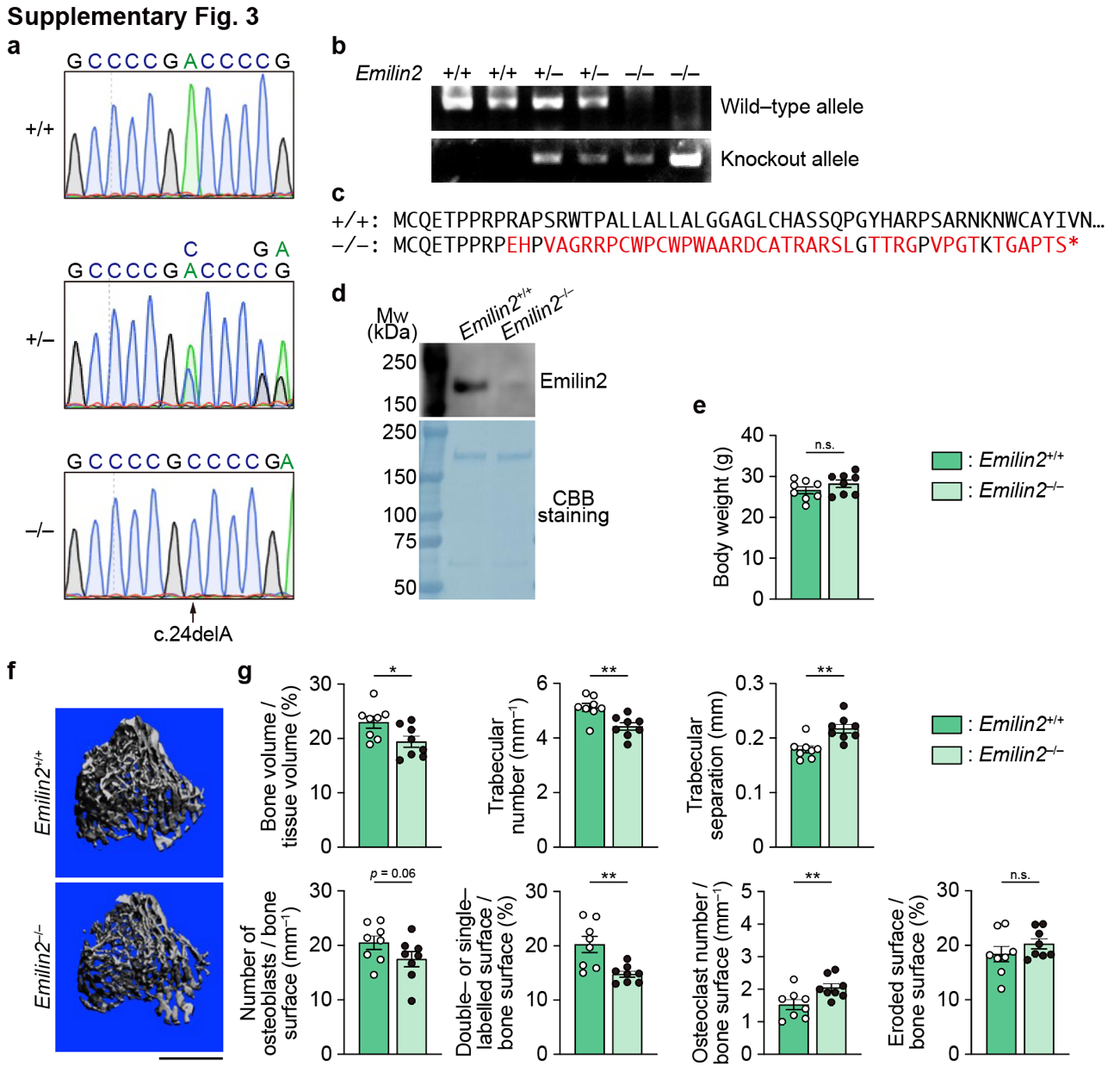
Supplementary Fig. 3** Generation of *Emilin2*^–/–^ mice. **a** Deletion of a single nucleotide in the *Emilin2* gene. Adenine at 24 base pairs downstream from the first ATG was deleted from the genomic DNA. **b** PCR analysis for genotyping. **c** Emilin2 translation products of *Emilin2*^+/+^ and *Emilin2*^–/–^ mice. Different amino acids and stop codon are highlighted in red. *: stop codon. **d** Western blotting and coomassie brilliant blue (CBB) staining images of the conditioned medium of *Emilin2*^+/+^ or *Emilin2*^–/–^ macrophages. **e** Body weight of *Emilin2*^+/+^ and *Emilin2*^–/–^ mice. **f** Representative 3D images of cancellous bone of proximal tibia, reconstructed from micro–CT images. Scale bar, 1 mm. **g** Bone parameters obtained by micro–CT analyses and histomorphometric analyses (n = 8). Statistical analyses were carried out using Student’s *t* test or Welch’s *t* test. Error bars show the mean ± s.e.m. **p* < 0.05; ***p* < 0.01; n.s., not significant.
