# Supplementary material for: Emilin2 marks the target region for mesenchymal cell accumulation in bone regeneration
Source: Inflamm Regen. 2024 Jun 3;44:27. doi: 10.1186/s41232-024-00341-6 (PMC11145771; doi:10.1186/s41232-024-00341-6)
Supplement: Supplementary file 4 — Additional file 4:. Fig. S4. Emilin2 is expressed upon acute inflammation. a The correlation between EMILIN2 and M1/M2 macrophage marker genes in human cells. b EMILIN2 expression by CD14+ human monocytes upon stimulation. Data was obtained from public databases, FANTOM5 human promoterome (https://fantom.gsc.riken.jp/5/). c mRNA expression of Emilin2 in mouse bone marrow after bone injury. d mRNA expression of Egfr in mouse bone marrow after bone injury. e The number of CD11b+ cells in the bone injury tissue of Emilin2+/+ and Emilin2–/– mice (n = 4). For the comparison of the 2 groups, statistical analyses were carried out using Student’s t test or Welch’s t test. For the multiple comparison, one-way ANOVA test followed by Dunnett’s test was carried out. Error bars show the mean ± s.e.m. n.s., not significant. FPKM: fragments per kilobase of exon per million reads mapped. [file 41232_2024_341_MOESM4_ESM.docx]

**
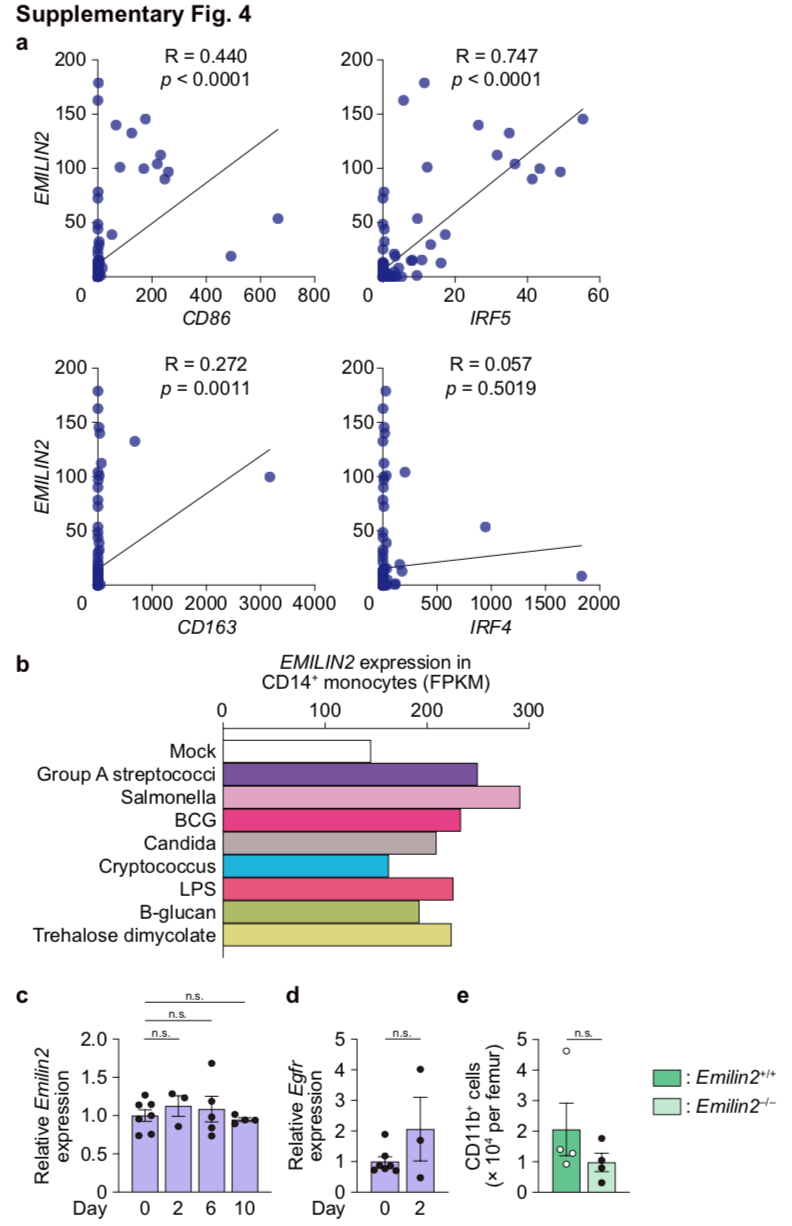
Supplementary Fig. 4** Emilin2 is expressed upon acute inflammation. **a** The correlation between EMILIN2 and M1/M2 macrophage marker genes in human cells. **b** *EMILIN2* expression by CD14^+^ human monocytes upon stimulation. Data was obtained from public databases, FANTOM5 human promoterome (https://fantom.gsc.riken.jp/5/). **c** mRNA expression of *Emilin2* in mouse bone marrow after bone injury. **d** mRNA expression of *Egfr* in mouse bone marrow after bone injury. **e** The number of CD11b^+^ cells in the bone injury tissue of *Emilin2*^+/+^ and *Emilin2*^–/–^ mice (n = 4). For the comparison of 2 groups, statistical analyses were carried out using Student’s *t* test or Welch’s *t* test. For the multiple comparison, one–way ANOVA test followed by Dunnett’s test was carried out. Error bars show the mean ± s.e.m. n.s., not significant. FPKM: fragments per kilobase of exon per million reads mapped.
