# Supplementary material for: Emilin2 marks the target region for mesenchymal cell accumulation in bone regeneration
Source: Inflamm Regen. 2024 Jun 3;44:27. doi: 10.1186/s41232-024-00341-6 (PMC11145771; doi:10.1186/s41232-024-00341-6)
Supplement: Supplementary file 5 — Additional file 5: Fig. S5. Local administration of clodronate liposome reduces macrophages in the bone-surrounding tissue but not in the bone marrow. a mRNA expression of macrophage marker genes in the bone-surrounding tissue. b mRNA expression of macrophage marker genes and Emilin2 in the bone marrow (n = 4 and 5, respectively). Statistical analyses were carried out using Student’s t test or Welch’s t test. Error bars show the mean ± s.e.m. *p < 0.05; ***p < 0.001; ****p < 0.0001; n.s., not significant. MΦ depletion: macrophage depletion. [file 41232_2024_341_MOESM5_ESM.docx]

**
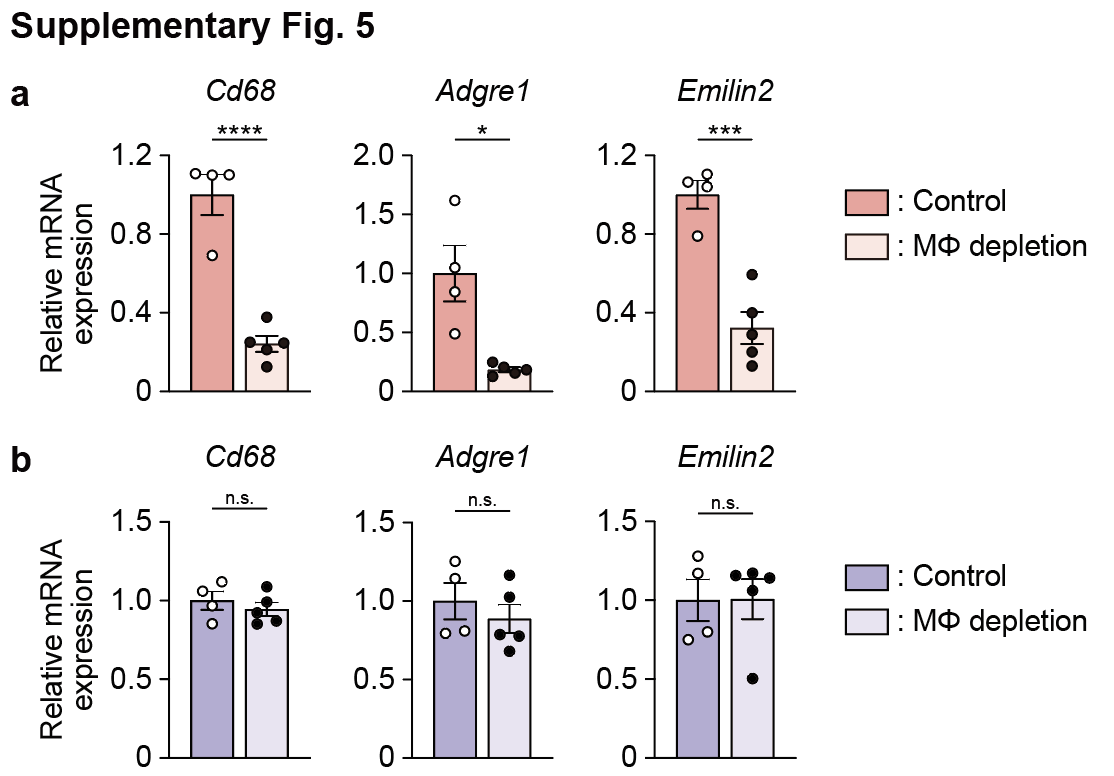
Supplementary Fig. 5** Local administration of clodronate liposome reduces macrophages in the bone–surrounding tissue but not in the bone marrow. **a** mRNA expression of macrophage marker genes in the bone–surrounding tissue. **b** mRNA expression of macrophage marker genes and *Emilin2* in the bone marrow (n = 4 and 5, respectively). Statistical analyses were carried out using Student’s *t* test or Welch’s *t* test. Error bars show the mean ± s.e.m. **p* < 0.05; ****p* < 0.001; *****p* < 0.0001; n.s., not significant. MΦ depletion: macrophage depletion.
